# Changing times, challenging times

**DOI:** 10.1111/hex.13177

**Published:** 2020-12-21

**Authors:** Carolyn A. Chew‐Graham

**Affiliations:** ^1^ School of Medicine Keele University Keele UK

Welcome to the final edition of Health Expectations 2020. It has certainly been an eventful year one which we will all look back as the year with *impact*. On topic, Dong and colleagues describe a discrete choice experiment and quantifying the general public's stated preference for the COVID‐19 vaccination programme. Their results provide policy‐makers internationally to identify individual and societal values for a good vaccination strategy. Asimakopoulou demonstrated that their study participants overwhelmingly believed that, as compared to people of their age and gender, they were somewhat or extremely unlikely to have accidentally infected people with COVID‐19 in the past, and to infect others or become infected themselves in the next month. They were also comparatively optimistic, but to a lesser extent, about their likelihood of getting hospitalized due to COVID‐19 and being admitted in an intensive care unit. We look forward to welcoming manuscripts reporting the experiences of people who have experienced COVID‐19.

This year, HEX has strengthened both the Editorial Board and the Editorial team. You can see the list of people who contribute to the smooth production of HEX at

https://onlinelibrary.wiley.com/page/journal/13697625/homepage/editorialboard.html

As always, this edition contains a number of papers employing qualitative research methods exploring clinical areas such as cardiovascular disease and focussing on age groups varying from young people to older adults and reflections on services (outpatient clinics and referral pathways). There is also a focus on patient‐centredness and engagement of patients in research—all topics which we believe are key for HEX.

To ensure we have the lay voice in the manuscripts we accept, we have added the following statement to Author guidelines ‘we require that authors include details of Patient and Public Involvement and Engagement in their research.’ Going forwards, if manuscripts do not include descriptions of lay involvement in the design or conduct of the study, or in data analysis or commenting on the meaning or the results, we will reject manuscripts without sending for peer review.

On the topic of peer review, all members of the Editorial team are finding it increasingly difficult to find reviewers for the excellent manuscripts we receive. We all absolutely understand that colleagues are working incredibly hard, particularly due to the impact of COVID‐19 and resulting restrictions, and are very grateful to all colleagues (especially those with clinical commitments) who do spare the time to provide reviews. Back in our own research teams, the Editors remind their colleagues to agree to review two papers for every one manuscript they submit. We would encourage senior academics to do the same in their teams.

Finally, the team at Wiley has been analysing how our audience uses HEX; it is apparent that, now we are completely online, very few people now read an issue ‘cover‐to‐cover’. We realize that the ‘Editorial briefings’ are not often read, and thus have decided to cease publishing them for regular issues, but we will still include Editorial briefings for ‘Special Issues’, with guest editors contributing to the articles. Do look out for the Mental Health Special Issue early in 2021.

I do hope you continue to contribute to, read, and enjoy manuscripts in HEX. Remember—*Health Expectations* aims to promote critical thinking and informed debate about all aspects of patient and public involvement and engagement (PPIE) in health and social care, health policy and health services research.

